# COVID-19 in Farm Animals: Host Susceptibility and Prevention Strategies

**DOI:** 10.3390/ani11030640

**Published:** 2021-02-28

**Authors:** Sachin Subedi, Sulove Koirala, Lilong Chai

**Affiliations:** 1 Faculty of Animal Science, Veterinary Science and Fisheries, Agriculture & Forestry University, Chitwan 44200, Nepal; sachinsubedi59@gmail.com (S.S.); sulovekoirala@gmail.com (S.K.); 2 Department of Poultry Science, College of Agricultural and Environmental Sciences, University of Georgia, Athens, GA 30602, USA

**Keywords:** Coronavirus, SARS-CoV-2, zoonotic, animals, vaccination, biosecurity measures

## Abstract

**Simple Summary:**

The outbreak of SARS-CoV-2 (also known as COVID-19) has caused pandemic diseases among humans globally so far. The COVID-19 infections were also reported on farm and pet animals, which were discussed and summarized in this study. Although the damage of COVID-19 has not been reported as serious as highly pathogenic avian influenza (HPAI) for poultry and African Swine Fever (ASF) for pigs on commercial farms so far, the transmission mechanism of COVID-19 among group animals/farms and its long-term impacts are still not clear. Prior to the development of the effective vaccine, the biosecurity measures (e.g., conventional disinfection strategies and innovated technologies) may play roles in preventing potential spread of diseases/viruses.

**Abstract:**

COVID-19 is caused by the virus SARS-CoV-2 that belongings to the family of Coronaviridae, which has affected multiple species and demonstrated zoonotic potential. The COVID-19 infections have been reported on farm animals (e.g., minks) and pets, which were discussed and summarized in this study. Although the damage of COVID-19 has not been reported as serious as highly pathogenic avian influenza (HPAI) for poultry and African Swine Fever (ASF) for pigs on commercial farms so far, the transmission mechanism of COVID-19 among group animals/farms and its long-term impacts are still not clear. Prior to the marketing of efficient vaccines for livestock and animals, on-farm biosecurity measures (e.g., conventional disinfection strategies and innovated technologies) need to be considered or innovated in preventing the direct contact spread or the airborne transmission of COVID-19.

## 1. Introduction

Coronaviruses (CoVs) belong to the Nidovirales order, which includes Coronaviridae, Arteriviridae, Mesoniviridae, and Roniviridae families [1]. The outbreak of SARS-CoV-2 (also known as COVID-19). COVID-19 is caused by SARS-CoV-2 (Severe Acute Respiratory Syndrome Coronavirus 2). This disease was firstly reported in December 2019 [2]. Bats are currently under consideration to be a probable origin of this virus [3]. COVID-19 has affected most of countries all around the world with 66.4 million confirmed cases and 1.52 million deaths as of 6th December, 2020 [4]. SARS-CoV-2 has been found to also infect animals and thereby creating a zoonotic potential [5,6]. The ongoing pandemic has spread fears among farmers who will be required to cull animals if their animals get infected by COVID-19 [7,8]. Therefore, the host susceptibility of this disease and the prevention measures knowledge are important for farmers as well as pet owners in current situation. The objectives of this study were to provide summarized information regarding farm and pet animals infected by COVID-19 in different countries or regions; (1) discuss potential spreading between animals and humans and animal test techniques, and (2) address how on-farm biosecurity measures (both conventional and innovated) may help to prevent the possible viruses spreading. 

## 2. Host Susceptibility for COVID-19

In a study regarding host susceptibility, Shi et al. performed inoculation of SARS-CoV-2 virus in various animals such as ferrets, cats, dogs, pigs, chickens and ducks [5]. Ferrets and cats were reported to be the most susceptible species and viral replication in these species were more pronounced. Pigs, chickens and ducks returned seronegative after virus inoculation in these animals in that study, which indicates these animals are less susceptible [5]. Another independent study concluded that chickens and pigs were not susceptible to SARS-CoV-2 [9]. A 17-year-old Pomeranian dog tested weakly positive for SARS-CoV-2 by PCR test (initial tests ruled out immune response); however, later tests proved that it was seropositive [10,11]. Data from several research studies indicate that dogs may not transmit SARS-CoV-2, but it is still unclear [12,13,14,15]. 

A study by Zhang et al. (2020) suggested that cats were infected by COVID-19 during outbreak in humans [16]. A research demonstrated that artificially inoculated cats with SARS-CoV-2 was able to transmit to other previously uninfected cats [17,18]. There has been handful of cases where humans were able to transmit this disease to their pet cats [19,20,21]. Ferrets exhibited virus replication and shed viruses in nasal discharges, saliva, feces and urine for up to 8 days [5,9,22]. Infection reported on mink farms in Denmark resulted in culling of millions of minks on many farms [23,24]. There is also a strong evidence for anthropozoonotic transmission of SARS-CoV-2 from minks [25]. Some rectal swabs from minks during screening returned positive for SARS-CoV-2 [6]. Table 1 shows the summarized information regarding reported COVID-19 on farm animals and pets. 

SARS-CoV-2 has been reported affecting pets with pre-existing diseases. This was reported in dogs that with a number of pre-existing diseases, including a grade II heart murmur, systemic and pulmonary hypertension, chronic renal disease, hypothyroidism and hyperadrenocorticism and the dog had got infection form 60-year-old owner who had developed symptoms of COVID-19 [26,29,30]. Histological findings of farm minks infected with SARS-CoV-2 revealed severe diffuse pneumonia with hyperemia and alveolar damage [6]. The latency period for SARS-CoV-2 is almost similar in humans and animals which ranges from 3–7 days to up to 14 days while the symptoms in animals are not certain, some developed dry cough with sneezing and lethargic signs [46]. Currently, the common diagnostic tests used to test animals for SARS-CoV-2 include virus neutralizing antibody test and reverse transcriptase polymerase chain reaction (RT-PCR) [37]. In virus neutralizing antibody test, blood will be collected and the serum will be separated for use in an in vitro assay to assess whether antibodies are inhibiting, or neutralize, the ability of a purified SARS-CoV-2 isolated to infect a permissive cell line [47].

## 3. Potential Prevention and Control Strategies

Preventive and control strategies for reducing spread of COVID-19 between humans are recommended by World Health Organization (WHO), government health departments, and health researchers, including personnel hygiene care, wearing a facemask, social distancing, temperature screening, early testing and report, and quarantine of peoples of suspected or infected individuals’ to mitigate human to human transmission and preventing further spread [4,48,49,50]. Temporary ban on wildlife trade was imposed in many countries following the outbreak of COVID-19 as the virus [51]. Proper regulatory mechanism is needed for wild animal trade as the live mammals acts as an intermediate host [52]. In order to mitigate the transmission of the virus and to evaluate the epidemics risk, it is recommended to focus on screening, identification, isolation, and characterization of coronaviruses present in wildlife species, especially in bats [51]. 

According to Astrid Iversen, a virologist at the University of Oxford, UK, who said due to rapid and uncontrollable spread of COVID-19 virus in minks, which makes animals a massive viral source that can easily infect people, culling of animals is probably essential [53]. However, there are a lot of animal welfare and animal rights concerns. Jannik Fonager, a virologist at Statens Serum Institute, said that the unchecked spread in mink also increases the opportunity for the virus to evolve [53]. In reference to the case of infected minks in the Netherlands, companion animals may also have capacity to spread COVID-19 to other people in the household or people being in close contact with the animals. Therefore, it is sensible for humans to avoid unnecessary contact with animals and should take care of basic sanitation measures when handling or caring for animals or animal products [54]. 

Rapid political responses and disease regulation play important role in emergence of zoonotic diseases like COVID-19 [55]. Mitigation measures such as reducing disease transfer from human to human, from animal to animal, between human and animals, protection of natural resources, and systemic policy change are important points to be considered for sustainable disease control [55]. Designating susceptible animal models using ferrets, cats, and macaques for the study of SARS-CoV and SARS-CoV-2 pathogenicity is critical for developing genetically modified animal models (induced models) for the prevention of COVID-19 [56]. One-health approach is very essential for prevention and control for the protection of both humans and animals. Overall, countries should have a one-health approach in their prevention and control strategy to protect both humans and animals from being infected, which can have a positive impact on prevention and control and, consequently, in the economy [57]. Boosting the immunity power in animals is major defense for COVID-19 until specific vaccines and medicines are available. Strategic plans for management, feeding, and health care programs is necessary for sustaining animal production [58]. 

**Figure 1:**
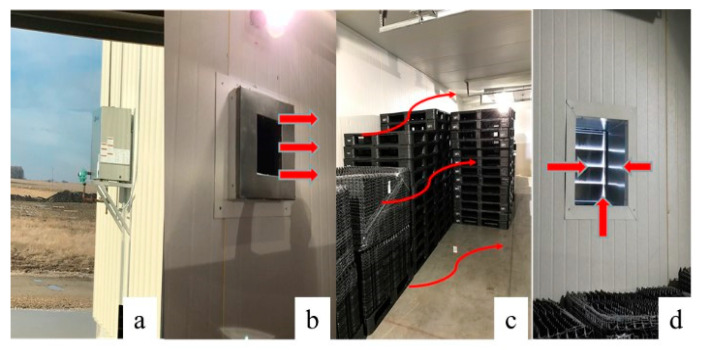
Heat treatment room (room dimension is 11.6 L × 3.9 W × 3 H m; (**a**)—heater, (**b**)—air outlet of heater, (**c**)—stacked pallets/flats, (**d**)—air outlet of the room) tested by Chai et al. [60] on a commercial poultry farm.

COVID-19 has yet caused disaster infection in commercial livestock and poultry as what the Highly Pathogenic Avian Influenza (HPAI) or African Swine Fever (ASF) has led for poultry and swine production [59,60]. However, the transmission mechanism of COVID-19 among group animals and farms are not well studied yet. Before the right vaccine is successfully developed and marketed, conventional and emerging measures of on-farm biosecurity may help prevent transmission of COVID-19 among farm animals. Conventional farm biosecurity measures include vehicles disinfection with liquid spraying, ultraviolet light, and shower-in and show-out for all farm staff and visitors. Emerging biosecurity measures such as heat treatment and electrostatic air filtration have been tested in the US in recent years. Scientists have developed a heat treatment method (i.e., heat room temperature to 60 °C for 8 h, Figure 1) for disinfecting egg transportation tools during outbreak of HPAI in the Midwest [60].

**Figure 2:**
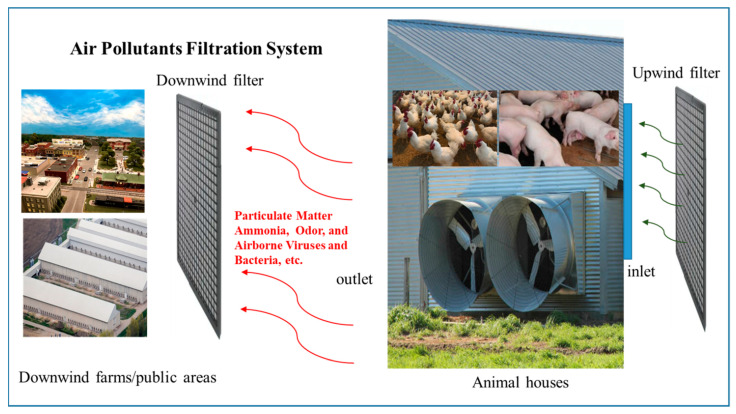
Diagram of air pollutants filtration system for animal houses.

In addition, air filtration system was tested for filtering the airborne dust at the inlet of poultry housing ventilation system to prevent potential airborne transmission of HAPI between farms or between animal houses on the same farm [61]. Figure 2 shows a potential air filtration system that may help filter polluted air entering or leaving the poultry houses. Those conventional and innovated biosecurity measures may be considered by farms if there are any outbreak of COVID-19 or other infectious animal diseases in the same region. 

## 4. Conclusions and Summary

COVID-19 has found to be efficiently replicate in cats, ferrets and farm minks. These are also capable of anthropozoonitic transmission of this disease. Therefore, precautions need to be taken if the immunocompromised owners are raising them. Currently, COVID-19 has not been found or reported infecting animals on poultry and pig farms. However, more research is needed to explore the host susceptibility and their capability of transmitting this disease in pig and chickens. It is critical to identify if pigs and chickens are susceptible to COVID-19 or the spread is mitigated by farm operations, because commercial farms usually have strict biosecurity measures such as disinfection on vehicles or shower-in and shower-out.

We have seen the progress in vaccine development for humans, but it is less clear whether animal vaccines are also making progress. Prior to the development of a successful vaccine, on-farm biosecurity strategies (e.g., conventional disinfection measures and innovative engineering technologies of electrostatic air filtration and heat treatment method) may play a role in preventing the spread of COVID-19 between commercial farms or animals.

## Figures and Tables

**Table 1:** Summarized reports of COVID-19 infections in animals based on reported time.

Farm Animals/Pets/Others	Animal Type	Country/Remarks	Date	Source
Pets	Dogs	Hong Kong, China	Feb, 2020	[26]
Farm Animals	Mink	Netherlands	April, 2020	[27]
Pets	Cats	Belgium	March, 2020	[28]
Pets	Cats	Hong Kong, China	March, 2020	[29]
Pets	Dogs	Hong Kong, China	March, 2020	[30]
Others	Tiger	India	April, 2020	[31]
Pets	Cats	USA	April, 2020	[19]
Others	Tiger, Lion	USA	April, 2020	[32]
Farms animals	Mink	The Netherlands	April, 2020	[33]
Pets	Cats, Dog	The Netherlands	May, 2020	[34]
Pets	Cats	Germany	May, 2020	[35]
Pets	Cats	France	May, 2020	[36]
Pets	Cats	USA	June, 2020	[37]
Pets	Dogs	USA	June, 2020	[37]
Pets	Cats	USA	July, 2020	[37]
Pets	Dogs	USA	July, 2020	[37]
Farm animals	Mink	Spain	July, 2020	[38]
Pets	Cats	USA	August, 2020	[37]
Pets	Dogs	USA	August, 2020	[37]
Farm animals	Mink	USA	August, 2020	[37]
Pets	Cats	USA	September, 2020	[37]
Pets	Dogs	USA	September, 2020	[37]
Farm animals	Mink	USA	September, 2020	[37]
Farm animals	Mink	Denmark	September, 2020	[39]
Pets	Cats	USA	October, 2020	[37]
Pets	Dogs	USA	October, 2020	[37]
Farm animals	Mink	USA	October, 2020	[37]
Others	Tiger	USA	October, 2020	[37]
Farm animals	Mink	Italy	October, 2020	[40]
Farm animals	Mink	USA	November, 2020	[37]
Others	Tiger	USA	November, 2020	[37]
Farm animals	Mink	Denmark	November, 2020	[24]
Farm animals	Mink	Greece	November, 2020	[41]
Farm animals	Mink	Sweden	November, 2020	[42]
Farm animals	Mink	France	November, 2020	[43]
Farm animals	Mink	Lithuania	November, 2020	[44]
Others	Lion	Spain	December, 2020	[45]

## Data Availability

Not applicable.
